# Public Health Response Systems In-Action: Learning from Local Health Departments’ Experiences with Acute and Emergency Incidents

**DOI:** 10.1371/journal.pone.0079457

**Published:** 2013-11-13

**Authors:** Jennifer C. Hunter, Jane E. Yang, Adam W. Crawley, Laura Biesiadecki, Tomás J. Aragón

**Affiliations:** 1 Center for Infectious Diseases and Emergency Readiness, School of Public Health, University of California, Berkeley, California, United States of America; 2 San Mateo County Health System, Division of Public Health, Policy and Planning, San Mateo, California, United States of America; 3 National Association of County and City Health Officials, Washington, District of Columbia, United States of America; 4 San Francisco Department of Public Health, San Francisco, California, United States of America; Inserm & Universite Pierre et Marie Curie, France

## Abstract

As part of their core mission, public health agencies attend to a wide range of disease and health threats, including those that require routine, acute, and emergency responses. While each incident is unique, the number and type of response activities are finite; therefore, through comparative analysis, we can learn about commonalities in the *response patterns* that could improve predictions and expectations regarding the resources and capabilities required to respond to future acute events. In this study, we interviewed representatives from more than 120 local health departments regarding their recent experiences with real-world acute public health incidents, such as infectious disease outbreaks, severe weather events, chemical spills, and bioterrorism threats. We collected highly structured data on key aspects of the incident and the public health response, particularly focusing on the public health activities initiated and community partners engaged in the response efforts. As a result, we are able to make comparisons across event types, create response profiles, and identify functional and structural response patterns that have import for future public health preparedness and response. Our study contributes to clarifying the complexity of public health response systems and our analysis reveals the ways in which these systems are *adaptive* to the character of the threat, resulting in differential activation of functions and partners based on the type of incident. Continued and rigorous examination of the experiences of health departments throughout the nation will refine our very understanding of what the public health response system is, will enable the identification of organizational and event inputs to performance, and will allow for the construction of rich, relevant, and practical models of response operations that can be employed to strengthen public health systems.

## Introduction

As part of their core mission, public health agencies attend to a wide range of disease and health threats, including those that require routine, acute, and emergency responses. In recent years, the 2001 anthrax attacks, the emergence of Severe Acute Respiratory Syndrome (SARS), the extraordinary destruction caused by Hurricanes Katrina and Sandy, and the pandemic from the novel H1N1 influenza virus have provided vivid examples of how natural and man-made phenomena can wreak havoc on the health and well-being of a community. Public health agencies have received increased attention and visibility following these events, which have been met with public investments in preparedness, as well as heightened expectations of the public health system’s ability to prevent, detect, and contain health threats to communities [1], [2].

As expectations have expanded, the need to strengthen public health systems’ capacity and capabilities to respond to any hazard has been at the center of many policy discussions [1], [2]. However, the evidence base for how to achieve this priority has lagged behind. There is still little agreement on how to measure, let alone improve, public health response performance [3], [4]. A number of challenges have been cited as barriers to research advancement in this field, including: the infrequent nature of large-scale public health emergencies [3], [5]–[7], the heterogeneity of emergency events and of public health delivery structures [3], [6], the challenges with access to incident leadership during real-world emergencies [7], the limited ability for standardized surveys to measure complex agency and system processes [6], and the difficulty of identifying a comparison group or constructing a counterfactual of what might have occurred if particular public health interventions had not taken place [8], [9]. As a result, outside of statistical modeling, researchers have often been limited in their use of statistical methods to test hypotheses, reach generalizable conclusions, and isolate factors that are likely to have the greatest impact on response capacity [8], [10]. Additionally, because catastrophic events are infrequent, the majority of the measurement literature in this field has focused on preparedness rather than on response -- on identifying and measuring the inputs to preparedness rather than the variations in response performance. What is known about public health emergency response is largely derived from simulated emergencies (e.g. exercises or drills), with a primary focus on bioterrorism or pandemic influenza [3]. By relying on an evidence base that draws from a narrow set of threats, we run the risk of overemphasizing the capabilities and resources required for those incidents while neglecting those that may be essential in other scenarios [11]. Furthermore, simulated emergencies introduce artificialities that do not reflect real-world response situations [12].

This study attempts to overcome these limitations and to inform agency preparation and performance by implementing a novel approach. First, we concentrate on characterizing responses to real-world events rather than preparedness and response efforts for hypothetical scenarios. Second, we broaden the case material to include acute public health events, not just disasters. And third, we compare response features across incidents rather than identifying lessons learned from single isolated incidents. The methodological basis for this approach is that while each event is unique, the number and type of response activities are finite; therefore, through comparative analysis, we can learn about commonalities in the response patterns that could improve understanding of the resources and capabilities required to respond to future acute events.

The purpose of this study was twofold: (1) to test this novel approach, and (2) to describe public health agency response patterns to a diversity of acute events. For this study, we collected highly-structured data on more than 120 real-world acute incidents, representing the broadest examination of events that have stressed the local public health system in the United States. By pooling data across diverse incident contexts and types, we increase the number of opportunities for learning [13]–[15]. This study serves as a starting point for the development of evidence-based forecasts of public health system response behavior that will help shape researchers’ and practitioners’ expectations for public health activity during urgent events and identify situations in which a governmental public health response has deviated from these expectations. Such deviations or “surprises” can provide opportunities to improve and update our understanding of response performance by pointing either to a lack of sophistication in our predictive models, adaptive response behaviors or promising practices that could be applied in other situations to beneficial effect, or unnecessary variation associated with inefficiencies that may affect the health of a community or the reputation of public health agencies.

Adopting the maxim that “all emergencies are local,” we focused this research on describing the public health response systems from the perspective of the local health department. We examine three domains through structured interviews with public health authorities involved in response efforts, including: (1) key characteristics of the acute event context, (2) the number and type of public response activities initiated using the CDC Public Health Preparedness (PHEP) Capabilities as an organizing framework, and (3) the number and type of organizations contributing to the public health response activities. The domains selected for this investigation were informed by a study of the organization and delivery of local public health services during normal operations by Mays *et al*. (2009), which employed similar measures in the expectation that they could reasonably be expected to influence performance and outcomes [16], [17]. We view these response measures as intermediate outcomes between an exposure (i.e. the urgent event) and the final outcomes of interest (e.g. illnesses, disabilities, deaths) [12].

## Methods

### Study design

This research uses a mixed-methods approach. Quantitative and qualitative data on urgent event and response characteristics were collected through structured telephone-based interviews with health department representatives using a retrospective cross-sectional design.

### Study population

**Selection criteria and sampling design.** The National Association of City and County Health Officials’ (NACCHO) 2010 National Profile of Local Health Departments (Profile of LHDs) was used to identify a sampling frame of 856 local health departments that serve a population of at least 50,000 individuals [18]. A total of 354 local health departments were recruited for participation, including: (a) all 171 local health departments that had responded to a Profile of LHD survey question indicating that their agency had responded to an “all-hazards emergency” between January 2009 and late 2010; (b) a random sample of 169 local health departments from the remaining sampling frame, using a probability-proportional-to-size sampling strategy; and (c) a convenience sample of 14 local health departments included in the pilot phase, with whom the researchers either had a personal connection or had learned about their involvement in incidents through an online disease outbreak alerting system, HealthMap.org [19].

To be eligible for participation, recruited health departments had to self-report that their agency had responded to an “urgent” event in recent history, defined as an event “whose scale, timing, or unpredictability overwhelmed or threatened to overwhelm routine capacity” [20]. Simulated emergencies and events related to the 2009 H1N1 influenza pandemic were excluded. Additionally, the representative(s) volunteering to participate in an interview had to indicate that they were generally knowledgeable about the overall public health response to the selected event.

**Recruitment.** Study recruitment and data collection proceeded in rounds, starting in March 2012 and ending in October 2012. In the initial rounds of recruitment, study invitations were emailed to preparedness coordinators and health officers for selected LHDs. Through follow-up phone calls with these health department representatives, we learned that personnel in different functional roles, specifically communicable disease control staff or epidemiologists, would be the best informed about the overall response to an infectious disease event. As a result, after approximately one-quarter of our sample had been recruited, we shifted our outreach strategy and targeted either (1) the preparedness coordinator, or (2) the communicable disease director or epidemiologist, in an effort to identify a range of infectious and non-infectious disease events. Each round of recruitment lasted six weeks, during which, individuals were sent the initial email invitation and study description, a reminder postcard by mail, three email reminders, and a telephone or voicemail follow-up. Individuals were also informed that they could forward the invitation to another person within the health department who might be better positioned to participate, and that multiple people could participate in a single interview.

As an incentive for participation, all participants were offered a customized report that would summarize their interview and provide a comparison to other de-identified health department(s) that had participated and discussed a similar event. Participants were also entered into a raffle for the chance to receive monetary prizes in the form of public health preparedness books.

### Measurements and Instrument

**Instrument.** Interviews were conducted by phone using a structured interview tool, which included questions related to the three primary research domains, including: (1) key characteristics of the acute event context, (2) the number and type of public response activities initiated using the CDC Public Health Preparedness (PHEP) Capabilities as an organizing framework, and (3) the number and type of organizations contributing to the public health response activities. The questions and response options were iteratively developed and refined through testing with over 100 case studies reported in the peer-reviewed literature and further revised after pilot-testing with four local health departments.

Three interviewers (two primary and one backup) were extensively trained on the intent of each question in the instrument, administration protocols, and response coding. Any questions regarding the interpretation and coding of interview responses were discussed throughout the data collection period. After all of the interviews had been completed, each of the two primary interviewers reviewed the others’ completed data collection tools to ensure that coding decisions were consistently applied.

**Measures.** Event characteristics. Each event was characterized with respect to a number of contextual features, which were selected with the goal of building a common operational picture that could allow for meaningful comparison across disparate incidents. We hypothesized that public health systems are *adaptive* to the nature of the event and therefore would expect to observe differential activation (in both number and type) of the response functions and partners based on the type of incident. In contrast, a non-adaptive response system would engage similar functions and partners regardless of incident type. Accordingly, our predictor variable was the type of event. Each incident was assigned to one of six specific event type categories, as defined by the CDC Emergency Preparedness and Response website, including: *infectious disease outbreaks and incidents, natural disaster or severe weather events, bioterrorism events, mass casualty events, chemical emergency events, or radiation emergency events* [21]. For the purposes of conducting certain analyses within this investigation, these categories were further collapsed into two groups: *infectious disease events*, including bioterrorism and infectious disease outbreak events, and *non-infectious disease events*, comprising the remaining event types.

For all events, regardless of type, the following event details were summarized: the duration of the public health response, the number of individuals directly contacted to investigate illness or exposure, the number of probable or confirmed cases, the number of severe cases (requiring hospitalization or resulting in death), and the number of persons receiving medical countermeasures as part of the public health response. Additionally, for each event, we recorded additional information data related to the scope of the event, such as the geographic locations affected, types of populations and community services affected by the event, and how frequently the health department responds to a similar event on the same scale as the one they selected for the interview.

**Public health response activities.** We used the CDC Public Health Preparedness Capabilities (PHEP Capabilities) framework and definitions as the basis for characterizing the public health activities carried out in response to the hazard [22]. The response activities are the first of our two primary outcome variables. The Capabilities framework identifies and defines 15 types of services that public health systems might be expected to deliver during emergencies. We deviated from this framework for the purpose of data collection in three key ways. First, we added four categories that emerged as important public health response activities through previous related work and pilot-testing but that are not emphasized in the PHEP Capabilities document. These categories included: environmental investigations, evacuation, consulting subject matter experts, and assessing medical and public health response capacity. Second, we eliminated the “preparedness” category from the list of public health response activities included in the interview since pilot-testing proved it was a confusing concept in the context of a specific response effort. Finally, we collected “other” activities that participants felt were important aspects of the response that had not otherwise been captured in the interview. Interviewers described each of the 19 response activity categories (14 original PHEP Capabilities, four additional categories, and an “other public health response activity” category) and asked participants to indicate whether any related activities were initiated during the response to their selected event. Additionally, participants were asked to identify which of the response activities were “absolutely necessary to the overall response.” A summary score was calculated by summing the total number of public health response activities initiated during an event (between 0 and 19 activities).

**Role of public health in the overall response.** In order to characterize the role of public health agencies in the event response, participants were asked to specify whether public health served in the lead role, joint-lead role with another responding agency, or supporting role.

**Organizational response partners.** The second outcome of interest is the public health response system, which we define as all entities who contributed to public health response activities for a given event. For each of the public health activities initiated during a response, participants were asked to identify the organizations and agencies that contributed to that activity, including their own organization. A list of 41 organization types was developed once all interviews were completed based on participants’ qualitative responses. The categorization of organizations was based on the descriptions of the public health system in the literature and expert opinion, using organizational function as the basis for classification [16], [23]–[25]. Three measures were developed from these data. The first measure, “any involvement”, is a dichotomous variable that indicates whether entities from each of the 41 organizational categories contributed to *any* of the 19 public health response activities. The second measure, “relative contribution”, is a weighted measure that summarizes, for each event, the number of response activities for which an organization type contributed, compared to the total number of response activities performed during that event. Therefore, for each event in which a specific organization type had any involvement, the “Relative Involvement” for an organization type was calculated as:



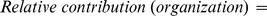


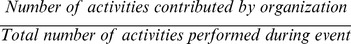


Additionally, a summary measure of the total number of organizational categories mentioned in the interviews was calculated (between 0 and 41 organizations).

**Alternate explanatory variables.** Because we expected that factors such as community context and local health department capacity also influenced the character of the public health response system, we also conducted an exploratory analysis to assess this relationship [16], [17]. Using data from the 2010 National Profile of LHDs, we assessed whether the number of response activities and partners varies by key characteristics of the health department, including: the population size served by a health department, health department expenditures, and number of full-time equivalent (FTE) staff [18]. Additionally, we examined whether the response activities and response partners vary based on the nexus of public health authority, which can be at the state or the local level.

### Statistical issues

Data recorded on paper-based interview tools were entered electronically into the web-based program, Qualtrics, using double data entry; the data were managed and analyzed using Stata 12 (StataCorp LP, College Station, TX) and merged with organizational data from the Profile of LHDs [18]. Distributions of event characteristics, response activities, and response partners were calculated and event-specific profiles were developed. For event and response measures, the differences between infectious disease and non-infectious disease events were assessed using *t* tests or chi-square tests, as appropriate, log-transforming data as necessary. A multiple linear regression model was employed to assess the association between organizational factors and response outcomes.

Based on our power analyses and primary research question, we decided to recruit at least 120 health departments. With this sample size, we expected to have power of 80 percent to detect significant differences of 25 percentage points or more between infectious disease and non-infectious disease events for the outcome response measures of interest.

### Ethics Statement

The protocols for this study were reviewed and approved by the Committee for the Protection of Human Subjects at the University of California, Berkeley, which determined that our research activities qualified for exempt status. Participants’ provided verbal informed consent to participate and to have the research interview audiorecorded, which was documented in the written record by the interviewer. This consent process is consistent with our Institutional Review Board's requirements for research with exempt status and with our approved research protocols. At this time, interview data are not available in a public repository.

## Results

### Sample demographics

**Figure 1:**
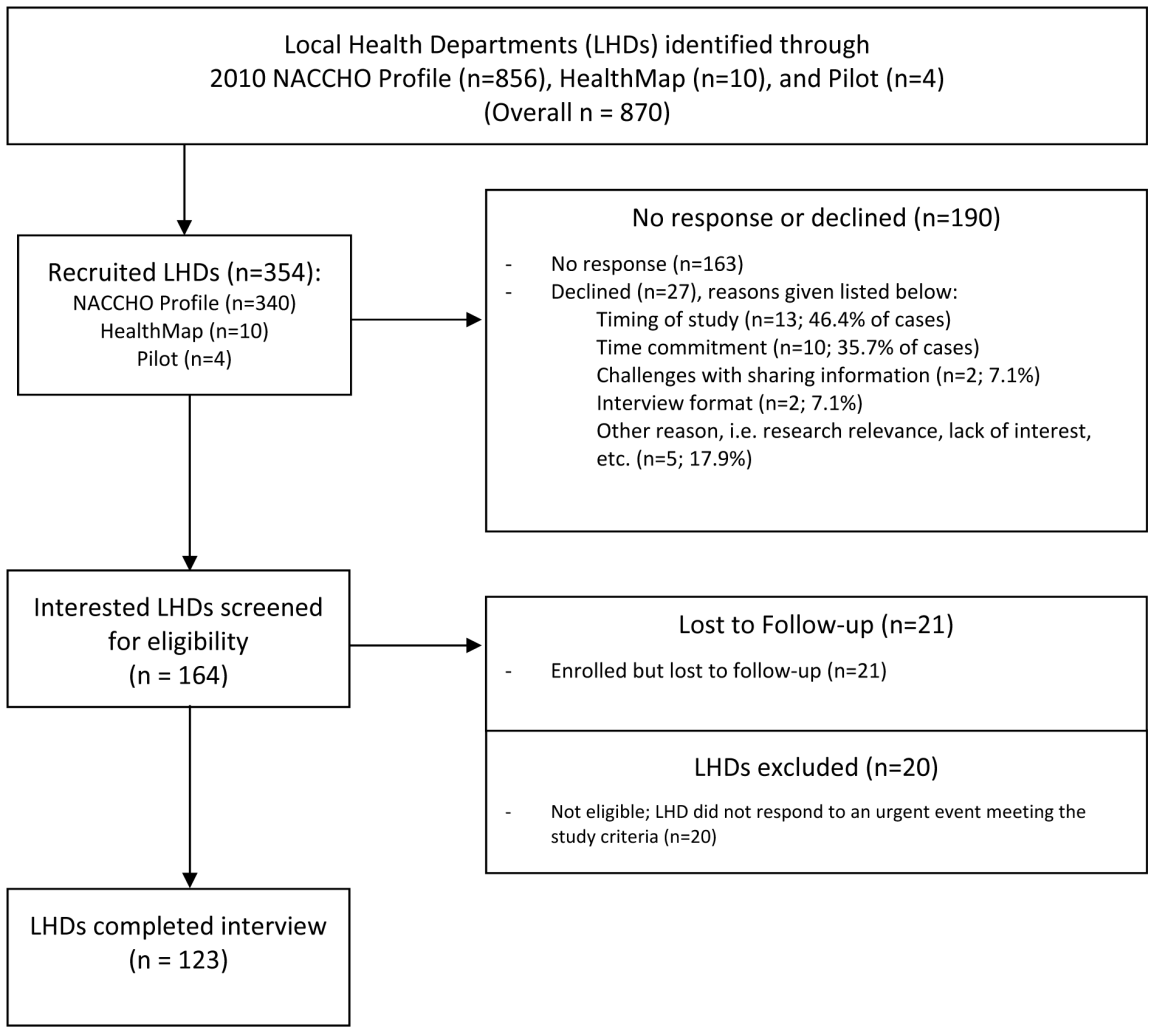
Recruitment Flow Diagram. This flow diagram summarizes the sampling and recruitment steps that resulted in the study population of 123 local health departments. Reasons for non-participation are provided, where possible.

Of the 354 recruited local health departments, participants from 123 health departments completed an interview, resulting in a 35% response rate. The 231 non-participating local health departments included agencies that: were not eligible because they did not have an urgent event that met study criteria (9% of non-participants), enrolled in the study but were lost to follow-up during the course of data collection (9%), declined to participate (12%), and provided no response to study recruitment requests (71%) (see **Figure 1**).

**Figure 2:**
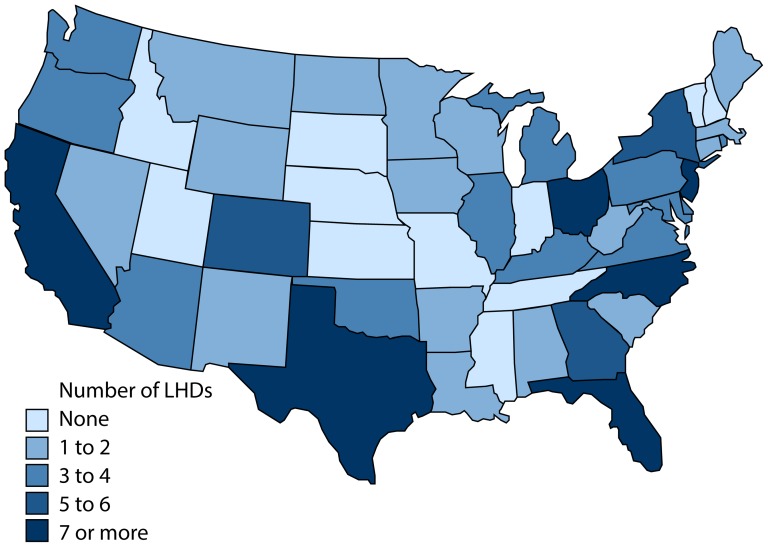
Geographic distribution of participating agencies, by U.S. state. This map shows the distribution of participating agencies across the United States. States with a greater number of participating local health departments (LHDs) are shaded a darker blue. States with the greatest number of participating health departments included California (12 LHDs), Ohio (8 LHDs), North Carolina (8 LHDs), Texas (7 LHDs), Florida (7 LHDs), and New Jersey (7 LHDs). Image developed using data from the National Weather Service and the SPMAP module for STATA 12 (College Station, TX: StataCorp LP) [33], [34].

Participants represented health departments in 38 of the 48 US states targeted for recruitment, with a diversity of community and public health agency characteristics (see **Figure 2** and **Table 1**). These agencies served populations from 50,000 to several million residents, reported annual expenditures ranging from $1 to more than $500 per capita, and had staffing levels between 10 and more than 1,000 Full-Time Equivalents. Nearly three-quarters of participants represented health agencies that operate as units decentralized from state health agencies (i.e. locally governed), with responsibility for a geographic jurisdiction defined by county boundaries. Overall, compared to non-participants, participating agencies were significantly more likely to serve a larger population, have more expenditure per capita, and have more FTEs (for all values, p<0.05).

**Table 1:** Characteristics of Participating Local Health Departments and Participants.

Continuous Variables	mean	median	min	max	Signif	National mean^1^
Population size (in thousands), n = 123	542	297	51	>2,000	*	294
Expenditures per capita (in U.S. dollars), n = 111	78	43	<10	>200	*	52
Number of Full Time Equivalent staff (FTE), n = 113	333	122	10	>1000	*	149
**Categorical Variables**	**n**		**% of Participants**		**% of LHDs Nationwide** 1	
**Governing authority**						
Centralized authority at the state	18		15		19	
Decentralized, authority at the local level	84		69		71	
Shared or mixed	19		16		10	
**Geographic area served by agency**						
City	14		12		10	
City-county	2		2		0	
County	85		70		69	
Multi-city	3		2		5	
Multi-county	17		14		16	
**Types of Services directly provided by agency**						
Comprehensive primary care services	20		17		18	
Any environmental health services	105		85		90	
**Position or title of participant(s)** 2						
Preparedness and Response	78		63			
CD Staff/Epidemiologist	47		38			
Environmental Health	12		10			
Health Director/Deputy	12		10			
Health Officer/Deputy	7		6			
Other	28		23			

Table 1 provides a descriptive summary of participants, including characteristics of the public health agency and agency representatives.

1 For local health departments serving a population of 50,000 individuals or more.

2 Participants could identify more than one position or title.

### Informant and interview characteristics

Interviews involved between 1 and 5 health department staff, such as preparedness and response coordinators or directors (63% of interviews), communicable disease staff, including directors, epidemiologists, and nurses (38% of interviews), health officers or directors (16%), and environmental health staff (10%) (see **Table 1**).

Interviews lasted nearly one hour, ranging from 27 minutes to 120 minutes. On average, interviews focusing on infectious disease events were significantly shorter than those focusing on non-infectious disease events (p<0.05).

### Event Characteristics

**Event details.** The types of events included in our study primarily involved infectious disease investigations and severe weather or natural disasters, with each constituting approximately 40 percent of the total. Our event set also includes incidents involving chemical exposures, misuse of prescription or illegal drugs, suspected or confirmed exposure to biological agents, radiation, mass casualties, technological emergencies (such as water or power outages), complex events (involving multiple causes), and anticipated mass-gatherings. Details on the types and frequencies of events are provided in **Table 2**.

**Table 2:** List of events.

Event Type	# of Events	Event detail (# of events)
**Infectious disease event**	51	Norovirus (9), Pertussis (7), Salmonellosis (6), Shiga toxin-producing Escherichia coli (STEC) (4), Tuberculosis (3), Hepatitis A (2), Measles (2), Meningococcal disease (2), Mumps (2), Bacillus cereus (1), Botulism (1), Campylobacteriosis and Guillian Barre Syndrome (1), Coliform bacteria (1), Cryptosporidiosis (1), Cyclosporiasis (1), Hantavirus pulmonary syndrome (1), Legionellosis (1), Lyme disease (1), Novel influenza A virus infections (1), Rabies-Animal (1), Shigellosis (1), Unknown Etiologic Agent (1), Varicella (Chicken pox) (1)
**Severe weather/Natural disaster**	45	Hurricane/Tropical Storm (16), Severe winter weather (7), Tornado (7), Flooding (5), Fire (5), Severe rain or wind storm/derecho (5)
**Chemical or drug event**	10	Designer drugs (Bath Salts/White Rush, Blueberry Spice) (2), Hydrogen Sulfide, Natural Gas, Mercaptans (1), Diesel Fuel And Lubricating Oil (1), Hydrogen Sulfide/Methane Gas (1), Pulverized Limestone (1), Deepwater Horizon - Crude Oil, tarballs (1), Isocyanate (1), Liquid Mercury (1), Lead, Arsenic (1)
**Event involving a biological agent (suspected or confirmed)**	6	White Powder Incident (Anthrax Suspected, Ruled Out) (3), Anthrax (Confirmed, From Natural Source) (1), Biowatch Actionable Result (Agent Not Named, Confidential) (1), Ricin (1)
**Radiation event**	4	Iodine-131, Cesium-134, Cesium-137 (3), Strontium-82 And Strontium-85 (1)
**Mass casualty event**	2	Explosion (1), Plane crash (1)
**Technological emergency**	2	Mechanical failure at water treatment plant (1), Transformer fire (1)
**Anticipated event**	2	Planned mass gathering (1), Displaced persons from natural disaster/severe weather (1)
**Complex event**	1	Displaced persons from natural disaster/severe weather & infectious disease outbreak (cholera) (1)
**Total**	123	

This table summarizes the total number of events within each event type. The number of events for each sub-category (e.g. number of urgent events involving the disease pertussis) is shown in parentheses.

When informants were asked how frequently their health department responds to a similar event on the same scale as the incident they selected for the interview, more than half indicated that this was the only event of its kind in recent history (29% of events) or that something similar happens once every few years (29%). Other events occurred with a greater frequency, from one to two times per year (28%) to three or more times per year (13%).

**Other context and indicators of event severity.** The characteristics of the events included in our study vary widely (see **Table 3**). For example, the shortest response duration was approximately five hours, in the event of a white powder incident, while the longest response lasted multiple years in the event of the Deepwater Horizon oil spill. The number of individuals within the community contacted by health departments and their partners to assess illness or exposure ranged from 0 to 11,000 individuals, resulting in the identification of a mean of 37 confirmed or probable cases per event. Overall, a mean of three cases resulted in hospitalization or death. A t-test comparison of the log-transformed variables, *duration of response* and *number of probable or confirmed cases*, revealed that infectious disease events involved significantly more cases (p<0.05) than did non-infectious disease events.

**Table 3:** Event Characteristics.

Event Characteristics	# of events	mean	sd	min	median	max	Signif.
**Duration of response (in days)**	(123)	64	125	0.2	18	854	**
**Number of individuals contacted to investigated illness or exposure**	(96)	756	2,111	0	80	11,000	
**Number of probable or confirmed cases**	(90)	37	82	0	7	565	*
**Number of severe cases (number of hospitalizations or deaths)**	(106)	3	7	0	0	51	
**Number of individuals who received prophylaxis**	(27)	1,253	2,376	0	161	10,240	

This table summarizes key event characteristics, including: duration of response time, number of individuals contacted to investigate illness or injury, number of probable or confirmed cases, number of severe cases, and number of individuals who received prophylaxis.

* Differences between infectious disease and non-infectious disease events significant at p < 0.05.

** Differences between infectious disease (excluding events involving a bioterrorism agent) and non-infectious disease events significant at p < 0.05.

All severe weather and natural disaster events directly or indirectly resulted in the disruption of community infrastructure or services, including water, sewage, electricity, telecommunications, roads or transportation, as well as the direct delivery of public health and medical services. On average, four types of services were disrupted in these severe weather events. With the exception of technological emergencies, other types of events rarely involved an interruption of community services other than those provided directly by public health, which were postponed or cancelled due to staff diversions for response activities in nearly a quarter of these events.

### Public Health Response Activities

**Figure 3:**
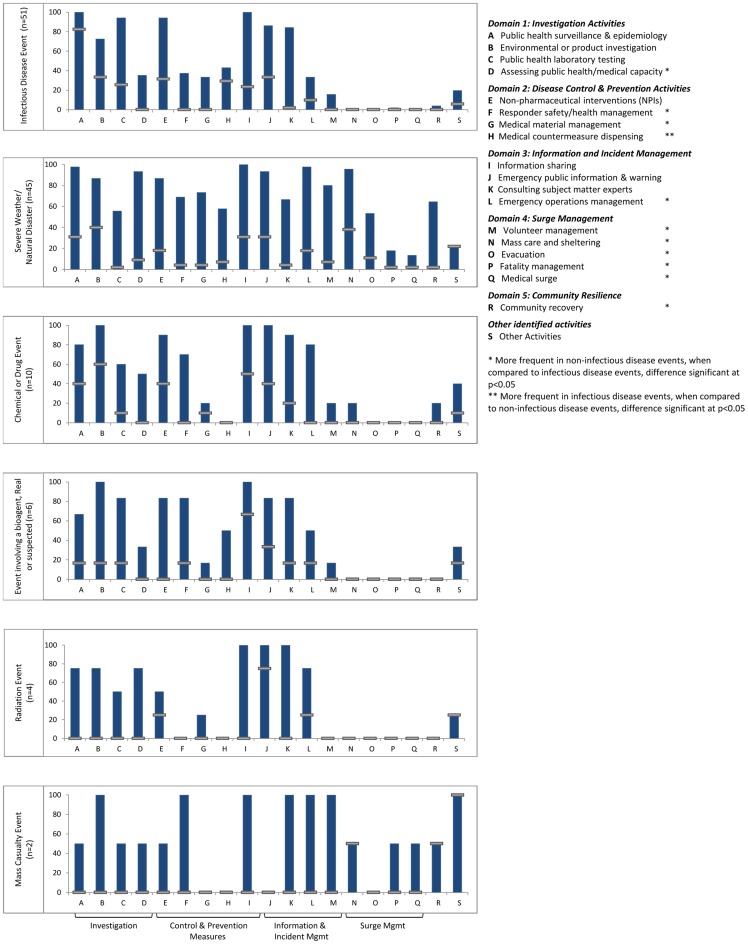
Response activity profiles, by event type. This figure shows the profile of response activities for each of six different types of events, displayed as separate bar charts. For a given event type, the blue vertical bars show the proportion of events that involved each of the 19 defined response activities. The horizontal gray bars provide the percent of events for which that activity was perceived to be “essential.” For example, within infectious disease events (top box), 100% of events involved epidemiology and surveillance (Activity A), and in 82% of events this activity was felt to be essential. The activities are ordered by five functional domains: investigation, disease control and prevention, information and incident management, surge management, and community resilience. Technological emergencies, complex events, and anticipated events are excluded from this figure due to small sample size.

In response to the urgent events included in our study, the number and type of public health activities initiated by response systems varied considerably (**Figure 3**). Of the 19 activity categories, urgent event response efforts involved between 3 and 18 types of activities, with a mean of ten activities per event. The response activities most commonly initiated were those related to: information sharing and management (100% of events), public health surveillance and epidemiology (98%), emergency public information and warning (89%), non-pharmaceutical interventions (88%), environmental or product investigation (82%), consulting subject matter experts (79%), public health laboratory testing (74%), and emergency operations management (65%).

Activity profiles for each of the six event categories (**Figure 3**) provide a summary of the frequency and distribution of response measures by event type. Response efforts for non-infectious disease events included a significantly greater number of response activities than infectious disease events, with a mean of 13 compared with 9 response activities, respectively. Only one type of activity, dispensing medical countermeasures, including vaccination and post-exposure prophylaxis, was more likely to occur during infectious disease events (p<0.05). In contrast, 11 different types of activities were significantly more common in non-infectious disease events, as shown in **Figure 3** with asterisks, (p<0.05). Because severe weather and natural disaster events constituted a majority of the non-infectious disease events, we conducted a sensitivity analysis to assess the robustness of the results. After excluding severe weather and natural disaster events, we found that infectious disease and non-infectious disease events did not differ significantly with respect to the number of response activities, and that the only differences in type of activity that persisted were: dispensing of medical countermeasures, which was still more common in infectious disease events; emergency operations management, volunteer management, and mass care and sheltering remained more common in the non-infectious disease events (p<0.05). Additionally, after excluding severe weather and natural disaster events, two new activities appeared to be more common to infectious disease events, including epidemiology/surveillance and laboratory testing (p<0.05).

Participants identified “other” public health activities that were carried out in the response to their event but were not captured by our pre-defined activity categories, including those related to: restoring community confidence after an event (e.g. community meetings, counseling individuals), enabling individuals to follow disease prevention and health promotion activities (e.g. obtaining food stamps after food disposal orders, providing financial assistance if ill or infected persons were excluded from work due to risk of disease transmission, providing housing for individuals removed from their homes), contributing to resource coordinating centers to help affected persons access needed health services and permits (e.g., food permits) from multiple agencies after an event, and assessing legal compliance and breaches of protocols.

**Essential Response Activities.** Our measure of *essential response activities,* shown as horizontal gray bars in **Figure 3**, provides a summary of activities that were perceived as “absolutely necessary” to the overall response. For the urgent events in our study, activities most commonly reported to be essential were: epidemiology and surveillance for ***infectious disease events***; environmental health and mass care and sheltering for ***severe weather events;*** environmental investigations and information management for ***chemical events;*** information management for ***events involving biological agents***; public information and warning for ***radiation events***, and “other” for ***mass casualty events,*** including patient transport and coordinating family assistance centers.

### Public Health Response System

**Figure 4:**
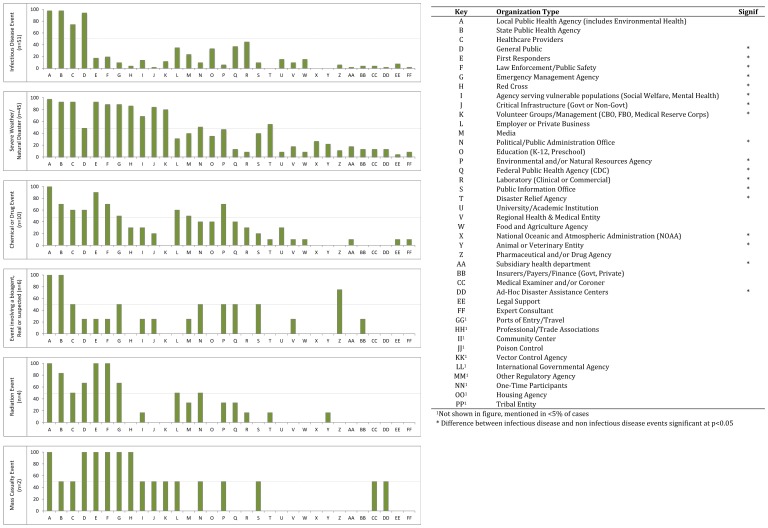
Public health response system profiles, by event type. Figure 4a shows the public health response system profile for each of six different types of events, displayed as separate bar charts, key provided in Figure 4b. For a given event type, the green vertical bars show the proportion of events that involved each of the 41 defined response partners. For example, within infectious disease events (top box), 98% of events involved local public health agencies (Organization Type A). The types of organizations are ordered based on the overall frequency with which they were mentioned, most frequent to least frequent, from left to right. A gray dotted line, at the 50% marker, is included in each bar chart to highlight those organizations involved in more than half of events of that type.

Of the urgent events included in our study, we found that public health response systems were comprised of 3 to 25 types of organizations, with a mean of 10 organizations (**Figure 4**). The types of organizations mentioned as contributors in more than half of the urgent event responses in our study included: local public health agencies, including environmental health (98% of events); state public health agencies (92%); healthcare providers (78%); members of the general public, including cases, contacts and family members of cases, and other individuals (70%); first responders, including emergency medical services, hazardous materials, and fire (58%); and law enforcement and public safety agencies (56%).

Overall, infectious and non-infectious disease events differed with respect to the numbers and types of public health system partners. The response systems for non-infectious disease events were comprised of significantly greater numbers of organization categories, with a mean of 13 versus 7 organization types respectively (p<0.05). We identified three organization types that were significantly more common in infectious disease events and 17 organization types that were more common in non-infectious disease events, shown in **Figure 4** (legend). Because the non-infectious disease category is dominated by severe weather and natural disaster events, we also conducted a sensitivity analysis to assess the robustness of our findings after excluding this type of event. We found that the difference in the number of response partners between infectious disease and non-infectious disease events remained significant after excluding severe weather events (p<0.05). However, only half of the previously observed differences in the types of response partners remained, including: general public, first responders, law enforcement, emergency management, American Red Cross, critical infrastructure, and laboratories (p<0.05). Additionally, after excluding severe weather and natural disaster events, two new differences in response partners appeared: involvement of ports of entry entities were more common in non-infectious disease events and involvement of state public health was common in infectious disease events (p<0.05).

For each incident, we also calculated the *relative contribution* or the proportion of initiated response activities to which a participating agency contributed. The vast majority of partner organizations contributed to a very limited proportion of the overall response activities. For example, volunteer organizations were primarily involved in mass care and sheltering or volunteer management activities, whereas the involvement of environmental or agricultural entities was mostly limited to environmental investigation and information sharing. Of forty-one organization types, only five contributed to more than ten percent of the response activities initiated during a response, including: local public health agencies, which contributed to a mean of 79 percent of response activities, state public health agencies (38% of response activities), healthcare providers (21%), first responders (14%), emergency management agencies (13%), and law enforcement agencies (11%).

**Role of public health in the response system.** Informants felt that public health played a “lead role in the overall response” to half of the events in this study, a joint-role in approximately one-third of events, and a supporting role in the remaining events. However, the public health role varied tremendously by the type of event, whereby public health was considered to play the lead role in 100% of the radiation and complex emergency events, 94% of infectious disease events, 33% of incidents involving a bioterrorism agent, 30% of chemical events, 9% of severe weather or natural disaster events, and none of the technological emergencies or anticipated events.

### Community and Public Health Agency Characteristics

Participants from health departments in which governmental authority is centralized at the state level, or where authority is shared between state and local entities, were significantly more likely to choose non-infectious disease events as the subject for the interview, compared to health departments that are decentralized from the state health department (p<0.05).

To assess the effect of agency characteristics on the response structure and function, multiple linear regression models were used to assess the relationship between the community and agency measures (predictor variables: size of the population served by a LHD, number of FTEs, and annual per capita expenditures) and response measures (outcome variables: number of organizations involved in the public health system response, number of response activities initiated during the response) controlling for the type of event (infectious disease versus non-infectious disease). Each of the six models included a single predictor and outcome variable, controlling for the type of event. We used the natural logarithm of each predictor variable and employed robust standard errors in the statistical models to minimize the effects of outliers and heteroskedasticity. Controlling for the type of event, only the models including the number of full time staff were significantly correlated with the number of response activities (F = 37.88, p = 0.005) and the number of partners (F = 57.88, 0.016), both showing a negative association. This correlation indicates an inverse correlation between the number of public health department staff and the number of organizations and the number of different types of activities activated during an event.

### Response reporting and dissemination

Eighty percent of participants indicated that their health department developed a report describing their response efforts. Approximately two-thirds of these health departments developed after-action reports, which were disseminated internally (66% of AARs), to contributing agencies (46%), or to the state health department (37%). In only 11 instances was a summary report widely disseminated, either in the peer-reviewed literature (4% of all events), in the *Morbidity and Mortality Weekly Report* (3%), or through the Department of Homeland Security Lessons Learned and Information Sharing web portal (LLIS, 2%).

## Discussion

This is the first study to systematically describe and analytically compare the response operations of local health departments and their community partners among such a large range of acute incidents. Public health representatives described their experiences responding to more than 120 incidents involving unusual clusters of illness, unexpected exposures to hazardous substances, or the sudden loss of infrastructure. Regardless of the character of the event, nearly every informant portrayed a situation that compelled his or her public health agency to work with a network of other organizations to take rapid action in an effort to mitigate, control, or prevent expected adverse health consequences, often in highly stressful and politically charged environments with demanding expectations for performance.

Typically, these local public health agencies are faced with restricted opportunities for learning from real-world urgent events. One reason is the infrequency with which these events occur for any given community. More than half of the health departments that we surveyed described an event that happens once every few years or an event that was the only one of its kind in recent history. A second reason is a lack of access to others’ experiences. Our findings show that less than ten percent of urgent public health incidents were summarized and disseminated to the outside world, likely due to a range of factors including time constraints and concerns over legal or political repercussions. Moreover, when incident summaries are actually shared, reports are so varied in structure and level of completeness that making comparisons across events and drawing parallels to one’s own experiences is very challenging [13]. Opportunities for learning from events faced by other health departments are further constrained by budget cuts, travel restrictions, and a funding environment that makes it difficult to justify activities that do not meet specific grant requirements [26]. As a consequence, for many types of events, health departments are limited in their own direct experiences and have almost no access to descriptions of the experiences of others. This environment stands in stark contrast to other organizations that are expected to perform reliably in high stress environments, such Naval aircraft carriers, where extensive field experience results in finely tuned expectations for behavior, or air traffic control systems, where the study and dissemination of lessons learned from near-miss incidents serves as a cornerstone for learning and improvement [27], [28]. Above all, this research demonstrates that it is possible to identify meaningful insights from a large set of real world events – insights that might not be evident from an examination of single isolated incidents – and that these lessons may have relevance in other public health settings and contexts.

### Complexity of public health response system

Our study contributes to clarifying the complexity of the public health response system, extending and expanding upon current system models [16], [23]–[25], [29]. First, our results demonstrate that public health response systems are *adaptive* to the nature of the threat. Our study response profiles reveal differential activation - in both number and type - of functions and partners based on the type of incident. For example, we found that the public health response to severe weather events involved a much larger and more diverse set of organization systems when compared to infectious disease events. Within the field of public organization theory, these non-infectious disease systems would be expected to elicit a number of predictable challenges to effective communication and coordination [30], [31]. With a more explicit recognition of these complex systems, researchers and practitioners may be able to better able to predict associated challenges, their consequences, and strategies for avoiding critical failure points during urgent events. Second, our study system profiles also provide an indication of the frequency and circumstances with which particular organizations might become involved in public health response activities – information that is of the greatest importance when developing and fostering relationships with potential partners in the community. We found that some entities are likely to take part in a public health response of any nature, including public health agencies, healthcare providers, and members of the general public, whereas many other organizations are either infrequently involved in public health responses or typically have a role only under specific event circumstances. The American Red Cross provides a good example of an organization that was almost universally active in our severe weather and mass casualty events, but rarely took part in infectious disease or bioterrorism events. Furthermore, the partner agencies described in our study, with few exceptions, lent their expertise or resources to a very limited proportion to the overall public health response activities. One interpretation of this finding is that only a fraction of response efforts will be salient to those organizations. Alternatively, this could suggest opportunities for expanded roles of organizations in response efforts. Lastly, we found that the role of public health varied tremendously by type of event. Public health departments were ten times more likely to serve in a lead role for infectious disease events compared to events involving severe weather. Recognizing these response patterns can have an impact on planning and exercising with partner agencies, particularly with respect to setting expectations and developing a mutual understanding about the roles and responsibilities of public health agencies in various situations, an issue that has repeatedly been recognized as an area needing improvement [3], [32].

When looking at health department characteristics, our findings suggest an inverse correlation between the number of full-time staff at a health department and the number of response organizations and activities activated during an event, after controlling for event type. While it might be expected that organizations with greater capacity would require less outside assistance, resulting in fewer organizations in the overall response system, it is somewhat surprising that we also observe a similar relationship with the number of response activities. One possible explanation is that health departments with fewer staff are required to work with a greater number of external organizations, and as a result, those organizations engage in a broader set of activities than a smaller response system.

### Using conceptual models of response to improve preparedness

Our study response profiles suggest a few predictable configurations common to public health response efforts. For example, while far from comprehensive or validated, our data identified three conceptual models of response. In the first model of response, most typical of infectious disease events, public health agencies serve in the lead role to the overall response; response activities and partners are more limited in number and type; the number of cases define event severity; and the epidemiology and surveillance function is considered most essential to the response. In the second model of response, most typical of severe weather and natural disasters, public health agencies serve in a joint or supporting role to the overall response; response activities and partners are more numerous and diverse; disruption to community infrastructure defines event severity; and environmental health and mass sheltering and care activities are considered most essential to the response. In the last model of response, typical of events involving chemical exposure or biological agents, public health agencies serve in a joint or supporting role to the overall response; response activities are moderate in number; response systems involve atypical partners; number of persons exposed defines event severity; and information and incident management activities are considered most essential to the response. An approach that uses “prototypical” models for response, such as this, could provide the basis for a new avenue of planning that builds on the strengths of those currently used. Like planning based on single scenarios (e.g. aerosolized anthrax), response models are grounded in concrete real-world incidents, making it easier to conceptualize the likely functional and structural aspects of a response. This allows for the development of detailed response protocols, which can be used to guide the training of staff and purchase of resources needed to test, implement, and improve these plans [11]. At the same time, the conceptual models are general enough that insights and skills gained from planning for one threat can be expected to be applicable, although not identical, to other hazards with a similar profile, increasing the efficiency of planning.

### Extension of CDC preparedness capabilities

We used a capabilities-based approach as an organizing framework for conceptualizing public health response activities. This planning model, based on an assumption that preparedness can be achieved by directing resources towards building, testing, and improving defined priority areas, is at the core of the CDC “preparedness capabilities” that were used to characterize the response activities described by our informants. Consequently, our results complement the PHEP Capabilities by highlighting the circumstances in which related activities or functions might have particular relevance in practice. Not surprisingly, our study finds that certain types of events were much more likely to elicit response activities related to particular capabilities, and that the frequencies with which capability-related activities were performed did not necessarily equate with how “essential” that capability was to the overall event. For example, the epidemiology and surveillance capability was almost universally activated. However, it was more likely to be considered “essential” for certain types of incidents, particularly infectious disease events. Linking our results to the CDC PHEP Capabilities framework may be of particular value to preparedness planners, for example, by guiding the selection of exercise scenarios that would be most likely to trigger activities related to the capabilities they seek to assess or improve. In addition to the original PHEP Capabilities, we also asked participants about four additional categories of activities that were identified through our previous research and pilot testing as (1) important and (2) of a different character than the PHEP Capabilities. These activities included: environmental or product investigation, consulting subject matter experts, assessing public health or medical capacity, and evacuation. While each of these proved to have relevance in certain contexts, environmental and product investigations stand out because informants mentioned these activities with such frequency, and considered these activities as essential in more than one-third of infectious disease, chemical, and severe weather incidents.

Currently, environmental investigations are folded into the “epidemiology and surveillance” capability; however, the resources, staffing, and partners required for these activities are quite distinct from those required for epidemiology and surveillance. We believe that under-specification of important response functions can have serious consequences, particularly in an era of scarce resources, in which health departments are often only able to direct their efforts to a limited number of preparedness improvements. Environmental investigations and other noted activities might be considered in future versions of the PHEP Capabilities or other discussions about what it means for communities to be prepared. Our informants also identified a variety of “other” public health activities carried out in the response to their events, that they felt were distinct from the PHEP Capabilities, and may also merit further attention.

Given the current fiscal and political environment, which increasingly demands accountability from the public sector, our findings may prove to be particularly informative. In the absence of strong empirical evidence, policy makers have relied on expert opinion and a very limited research base to guide the development of preparedness standards, guidance, and performance measures [6]. Our research demonstrates that the available literature is not representative of the urgent events that health departments face, and that the available descriptions of the public health system in-action do not reflect actual complexity. Our study strengthens this limited evidence base and we hope increases accountability and improves guidance, policy, and best practices in preparedness and response.

### Strengths

Our study benefits from three major strengths, including active event finding, a broad definition of urgent events, and the use of in-depth interviews as a data collection method. As a result, we were able to gain access to a number and diversity of urgent events that would not have otherwise been available. In fact, fewer than ten percent of the events included in this investigation were published in the peer-reviewed literature or other professional information-sharing web portals, confirming that the publicly available literature describes a very limited proportion of events experienced by LHDs. For example, only two percent of events were reported to the Department of Homeland Security’s Lesson Learned and Information Sharing (LLIS) web portal database, which is believed to contain a fairly comprehensive set of response summaries.

Second, our study adopted of a broad definition of urgent events, thereby expanding the available case material on public health responses. By pooling data across incidents with different contexts, we had a sufficient number to examine patterns, highlight commonalities and differences across events with different contexts, and generate hypotheses for future study. Third, because this is a fairly new field of research, the use of in-depth interviews for data collection was invaluable, as this method provided participants the opportunity to ask for clarification on questions and for interviewers to ask follow-up questions and to hear how health department representatives describe their response. These qualitative data, while not highlighted in our findings, influenced the insights we drew from the data. This study was also strengthened by the availability of organizational data, provided by the 2010 Profile of LHDs, which provided a sampling frame of local health departments, allowing us to better understand the representativeness of our sample and interpret our findings, and affording us the opportunity to examine how characteristics of a health department influence our outcomes of interest [18]. Finally, our approach also draws strength from the application of a systems-based and functions-based approach, both seen as essential features of high-quality research in this field [3]. By using the CDC PHEP Capabilities as a framework for conceptualizing public health activities, we hope to be able to contribute to the scientific literature in a way that is standardized, and thus allows for comparison with future research.

### Limitations and Next Steps

While our results describe the responses to a wide range of incidents, our method of event-finding did not draw from a sample that is statistically representative of all health departments or urgent public health incidents across the United States. Representativeness was not a cornerstone of our sampling goal; however, in order to appropriately interpret the findings of this study, we believe that it is important to recognize the ways in which our findings are not representative. First, while we were able to achieve the desired number of events for comparison, we had a fairly low overall response rate (35%). Based on the reasons for not participating provided by a subset of our non-participants, a significant proportion of health departments in this group may not have experienced an incident that met our study criteria. Therefore, we believe that the true response rate of *eligible* LHDs was considerably higher. Nonetheless, we recognize that participating agencies systematically differed from those who did not participate: they were significantly more likely to serve a larger population, have higher public health expenditures per capita, and have more full-time staff. Non-participants health departments’ capacity or inclination to participate may be related to a specific response profile that is underrepresented in our results. Second, we allowed participants to select a single event, which is one of many from which they could have potentially chosen. We do not claim to know anything about the events that were not selected; therefore, it is not possible to know how representative our set of events really is. We do know that the distribution of event types in our sample is similar to that found in other research [18]. Furthermore, certain types of events occur more frequently in our dataset, such as norovirus outbreaks and hurricanes. As a result, each event profile is disproportionately influenced by these more frequent events. Lastly, health departments that served a population of fewer than 50,000 individuals were excluded from our sampling strategy. The response system attributes of these health departments, which often have very limited staffing, warrant additional study.

Another limitation of this study is that we recorded only whether certain activities were initiated and which partners were involved in a response. We did not attempt to characterize the quality or appropriateness of those partners or activities. Additionally, we do not provide information on the organizational, inter-personal, leadership, training, or historical factors that likely influenced whether responding agencies considered response measures to be appropriate and actionable.

## Conclusion

In this study, we collect highly structured data on more than 120 real-world acute public health incidents. This is the first study to systematically describe and analytically compare the response operations of local health departments and their community partners during such a large range of acute incidents. As a result, we are able to make comparisons across events and to identify functional and structural response patterns that have relevance to public health practitioners and researchers. As an extension of this work, we recommend that future studies examine the types of events that were less commonly reported in our sample, including mass casualty and chemical events, and suggest continued use of standardized data gathering to ensure that future guidance, policy, and research is grounded in the best evidence learned through real-world events.
